# A meta-analysis of the incidence of malignancy in adult patients with rheumatoid arthritis

**DOI:** 10.1186/ar2404

**Published:** 2008-04-23

**Authors:** Allison L Smitten, Teresa A Simon, Marc C Hochberg, Samy Suissa

**Affiliations:** 1 Duke University School of Medicine, Duke South, Durham, NC, 27710 USA; 2 Global Pharmacovigilance and Epidemiology, Bristol-Myers Squibb Company, 311 Pennington-Rocky Hill Road; Hopewell, NJ, 08534 USA; 3 Division of Rheumatology and Clinical Immunology, University of Maryland School of Medicine, 10 S. Pine St., MSTF 8-34; Baltimore, MD, 21201 USA; 4 Division of Clinical Epidemiology, McGill University Health Centre, Royal Victoria Hospital; 687 Pine Ave West, R4.29; Montreal, QC, H3A 1A1 Canada

## Abstract

**Introduction:**

The risk of malignancies in patients with rheumatoid arthritis (RA) has raised some concern, particularly with immunosuppressive approaches to disease management.

**Methods:**

We conducted a systematic review of the literature and meta-analysis characterizing the associated risk of overall malignancy and four site-specific malignancies (lymphoma, lung, colorectal, and breast cancer) in patients with RA. A Medline search from 1990 to 2007 was conducted using specified search terms and predefined inclusion criteria for identification of relevant observational studies that provide estimates of relative risk of malignancy associated with RA. Study-specific estimates of the relative risk, as measured by standardized incidence ratios (SIRs) and estimated in comparison with the general population, were combined using a random effects model.

**Results:**

A total of 21 publications were identified, of which 13 reported the SIR for overall malignancy, 14 for lymphoma, 10 for colorectal, 12 for lung, and 9 for breast cancer. Compared with the general population, the overall SIR estimates suggest that RA patients have approximately a two-fold increase in lymphoma risk (SIR 2.08, 95% confidence interval [CI] 1.80 to 2.39) and greater risk of Hodgkin than non-Hodgkin lymphoma. The risk of lung cancer was also increased with an SIR of 1.63 (95% CI 1.43 to 1.87). In contrast, a decrease in risk was observed for colorectal (SIR 0.77, 95% CI 0.65 to 0.90) and breast (SIR 0.84, 95% CI 0.79 to 0.90) cancer. The SIR for overall malignancy was 1.05 (95% CI 1.01 to 1.09).

**Conclusion:**

Patients with RA appear to be at higher risk of lymphoma and lung cancer and potentially decreased risk for colorectal and breast cancer compared with the general population.

## Introduction

Rheumatoid arthritis (RA) is a chronic autoimmune disease that is also characterized by the presence of inflammation. Because of the immune pathways underlying its pathogenesis and what has generally been an immunosuppressive approach to disease management using traditional disease-modifying antirheumatic drugs (DMARDs), the risk of malignancies among RA patients has been of considerable interest. The characterization of this potential risk has become more relevant with the introduction of a new class of agents, biologic DMARDs. While these drugs act by directly modifying immunologic pathways involved in the pathogenesis of RA, it has been of concern that their use may be associated with an increased incidence of cancer. To better understand and interpret studies evaluating the risk associated with these agents, it is first necessary to determine the magnitude of any underlying risk of cancer that may already be present in patients with RA compared with the general population.

Data from several studies, reviewed by Chakravarty and Genovese [1], have suggested that there is no increase in the overall risk of cancer in patients with RA compared with the general population. However, accumulating evidence has suggested that the RA population may be characterized by changes in the relative risk of site-specific malignancies. Consequently, the objective of this study was to review the risk of four important site-specific malignancies (lymphoma, lung, colorectal, and breast cancer) in patients with RA in the recent published literature. In particular, this review focused on observational studies comparing the incidence of malignancy in patients with RA versus the general population since these may be expected to provide a realistic perspective on risk in the clinical setting.

## Materials and methods

To identify studies characterizing the risk of malignancy in patients with RA compared with the general population, a Medline search was performed using the search terms 'rheumatoid arthritis' combined with 'cancer', 'malignancy OR malignancies', 'neoplasm(s)', or 'lymphoma(s)'. The search covered the publication period from January 1990 to December 2007 and included only English language publications. Studies were eligible for inclusion if they fulfilled the following criteria: (a) observational-type study design (including prospective, retrospective, epidemiologic, database, survey, registry, cohort, and case-control), (b) more than 100 patients, (c) adult population, and (d) geographic regions including North America, South America, Europe, Australia, New Zealand, and Japan. Citations meeting the inclusion criteria were obtained and screened for the outcomes of interest, which included the observed incidence rates of total malignancy, lymphoma, lung, colorectal, and breast cancer in patients with RA compared with the expected incidence rates in the general population. Lymphoma was reported as Hodgkin or non-Hodgkin where available. The selection of studies for inclusion was made without regard to evaluation of specific RA management strategies. We attempted to avoid overlap by excluding studies for which updated manuscripts were available.

The preferred method of data presentation was the calculated relative risk compared with the general population, generally estimated as the age- and gender-adjusted standardized incidence ratio (SIR) and sometimes referred to as a standardized morbidity ratio. The SIR provides a point estimate of relative risk and is accompanied by a 95% confidence interval (CI). In situations in which SIRs were not specifically reported, they were calculated from the observed and expected incidence rates presented in the study (SIR = number of observed malignancies per number of expected malignancies), and a 95% CI was determined assuming that the frequency of observed cases followed a Poisson distribution. For the meta-analysis, summary estimates and 95% CIs were calculated based on the method of DerSimonian and Laird [2]. This method uses a random effects model that considers both within-study and between-study variation by incorporating the heterogeneity of effects in the overall analysis.

## Results

A total of 2,093 articles were identified using the defined Medline search criteria, and these titles and abstracts were screened to identify potentially relevant articles. A total of 106 publications were further analyzed for the presence of the inclusion criteria, and, of these, 21 publications from 16 diverse studies (Additional file 1) met all inclusion criteria [3-23]. These studies included population- and community-based RA cohorts that ranged from 144 to 76,527 patients and had mean follow-up times of 1 to 17.4 years. There was slight overlap in the patient populations included in two studies from Sweden [4,5,7].

Of these studies, 13 reported the relative risk for overall malignancy, 12 for lung cancer, 10 for colorectal cancer, and 9 for breast cancer. The relative risk of lymphoma was reported in 14 studies; 6 studies reported overall lymphoma, 10 reported non-Hodgkin lymphoma, and 8 reported Hodgkin disease.

**Figure 1:**
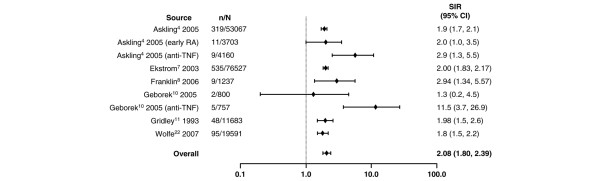
Relative risk of overall lymphoma in patients with rheumatoid arthritis (RA) compared with the general population. CI, confidence interval; n, number of malignancies; N, population size; SIR, standardized incidence ratio; TNF, tumor necrosis factor.

**Figure 2:**
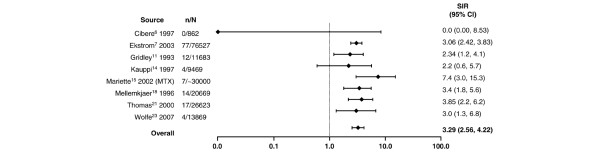
Relative risk of Hodgkin disease in patients with rheumatoid arthritis compared with the general population. CI, confidence interval; MTX, methotrexate; n, number of malignancies; N, population size; SIR, standardized incidence ratio.

**Figure 3:**
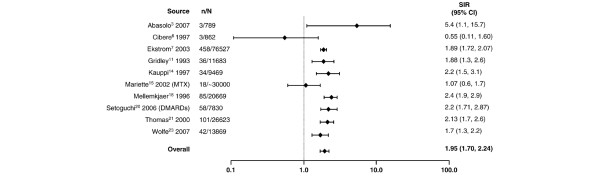
Relative risk of non-Hodgkin lymphoma in patients with rheumatoid arthritis compared with the general population. CI, confidence interval; DMARDs, disease-modifying antirheumatic drugs; MTX, methotrexate; n, number of malignancies; N, population size; SIR, standardized incidence ratio.

**Figure 4:**
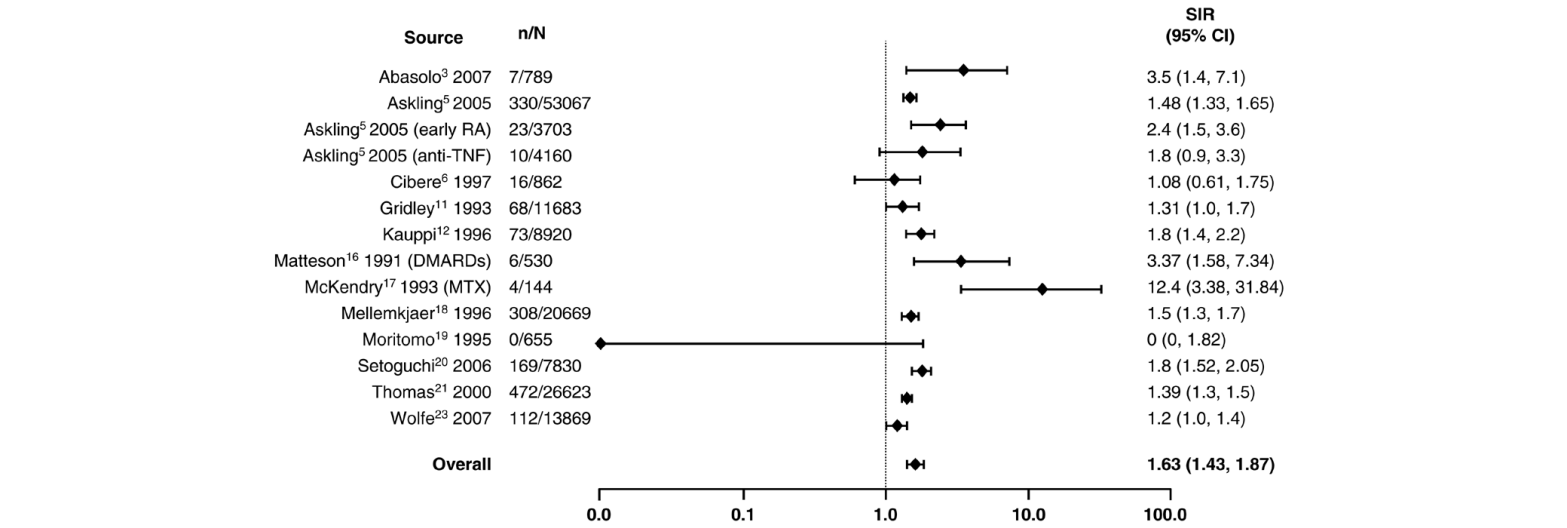
Relative risk of lung cancer in patients with rheumatoid arthritis (RA) compared with the general population. CI, confidence interval; DMARDs, disease-modifying antirheumatic drugs; MTX, methotrexate; n, number of malignancies; N, population size; SIR, standardized incidence ratio; TNF, tumor necrosis factor.

**Figure 5:**
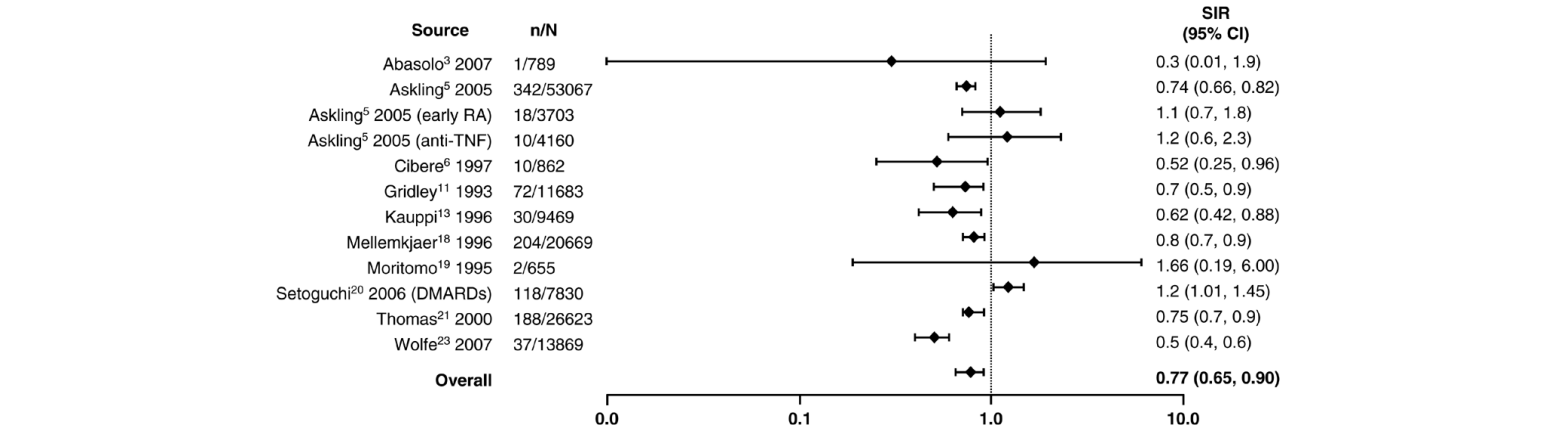
Relative risk of colorectal cancer in patients with rheumatoid arthritis (RA) compared with the general population. CI, confidence interval; DMARDs, disease-modifying antirheumatic drugs; MTX, methotrexate; n, number of malignancies; N, population size; SIR, standardized incidence ratio; TNF, tumor necrosis factor.

**Figure 6:**
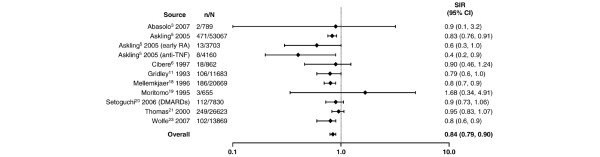
Relative risk of breast cancer in patients with rheumatoid arthritis (RA) compared with the general population. CI, confidence interval; DMARDs, disease-modifying antirheumatic drugs; MTX, methotrexate; n, number of malignancies; N, population size; SIR, standardized incidence ratio; TNF, tumor necrosis factor.

**Figure 7:**
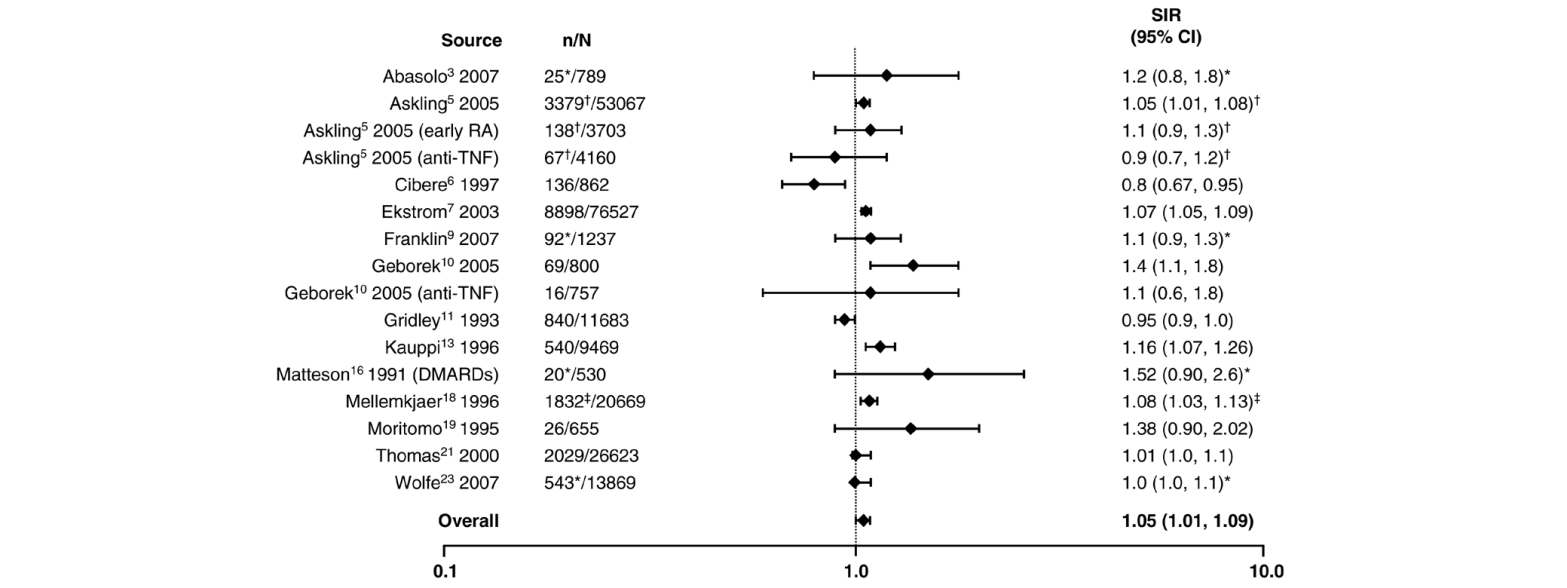
Relative risk of overall malignancies in patients with rheumatoid arthritis (RA) compared with the general population. *Excluding non-melanoma skin; ^†^all solid tumors; ^‡^excluding lymphatic and hematopoetic. CI, confidence interval; DMARDs, disease-modifying antirheumatic drugs; MTX, methotrexate; n, number of malignancies; N, population size; SIR, standardized incidence ratio; TNF, tumor necrosis factor.

All of the publications presented SIRs, but in two publications the SIRs were stratified by gender, necessitating recalculation of the SIRs for the combined population [12,21]. Figures 1 to 7 graphically present the SIRs and their 95% CIs from the individual studies for the site-specific malignancies and overall malignancy as well as the calculated point estimates and 95% CIs from the random effects models of the combined studies.

Compared with the general population, the highest risk of a site-specific malignancy in patients with RA was observed for lymphoma regardless of lymphoma type. Despite the wide range in relative risk, as shown in Figure 1 and based on the random effects model, RA was associated with an overall two-fold increase in lymphoma risk compared with the general population (SIR 2.08, 95% CI 1.80 to 2.39). A higher risk was observed for Hodgkin lymphoma (Figure 2) than for non-Hodgkin lymphoma (Figure 3), with SIRs of 3.29 (95% CI 2.56 to 4.22) and 1.95 (95% CI 1.70 to 2.24), respectively.

In addition to lymphoma, lung cancer was more frequently observed in patients with RA than in the general population (Figure 4). With the exception of four studies having outlying SIR values of 0 [19], 1.08 [6], 1.2 [23], and 12.4 [17], there appeared to be a cluster of SIRs that suggested an approximate 1.5- to 3.5-fold increase in the risk of lung cancer, a range that was supported by the random effects model which resulted in an SIR of 1.63 (95% CI 1.43 to 1.87). In contrast, the risk of colorectal cancer appeared to be somewhat reduced in patients with RA (Figure 5). For colorectal cancer, individual SIRs were generally less than 1, and the summary estimate using the random effects model resulted in an overall SIR of 0.77 (95% CI 0.65 to 0.90). Similarly, as shown in Figure 6, there appeared to be a slightly reduced risk of breast cancer associated with RA. With one exception, all the reported SIRs clustered just below risk parity with the general population, and the summary estimate was 0.84 (95% CI 0.79 to 0.90). The exception, an SIR of 1.68, was in a Japanese population and the authors state that they observed a generally higher risk of malignancies in Japanese women with RA than that reported in comparable Caucasian cohorts [19].

In general, the SIRs from the various individual studies were near parity for the risk of overall malignancies (Figure 7). The random effects model provided a summary estimate of 1.05 (95% CI 1.01 to 1.09).

Several of the studies examined the risk of malignancy in patients receiving biologic therapy [4,5,10,20,22,23]. In those studies that specifically evaluated the effects of tumor necrosis factor (TNF) antagonists on lymphoma risk, there was a higher risk in RA patients receiving anti-TNF therapy compared with the general population, with SIRs of 2.9 [4] and 11.5 [10]. Several of the studies that were included in our analysis presented odds ratios for lymphoma in RA patients who received anti-TNF therapy compared with RA patients who did not receive anti-TNF therapy, and none showed a statistically elevated risk associated with anti-TNF use [4,10,22]. Two studies presented SIRs for overall malignancy and neither was significant [5,10]. Askling and colleagues [5] presented SIRs for various solid tumors and reported no difference in patients with RA who received anti-TNF medication compared with the general population for lung and colorectal cancer, whereas the SIR for breast cancer was decreased (SIR 0.4, 95% CI 0.2 to 0.9). Wolfe and Michaud [23] computed odds ratios to evaluate the use of biologics in RA patients compared with non-use and did not find an association between these medications and overall malignancy (excluding non-melanoma skin), lung cancer, breast cancer, or colorectal cancer.

Two of the studies included in our analysis evaluated malignancy risk in an identified early RA population [4,5,8,9]. Askling and colleagues [4,5] found an increased risk of lymphoma and lung cancer and a decreased risk of breast cancer in patients with early RA compared with the general population; they found no association with all solid tumors or colorectal cancer. Franklin and colleagues [8,9] observed no increase in overall malignancy excluding non-melanoma skin cancer but saw an increase in lymphoma in their early RA population.

## Discussion

The data reported here suggest that, although there is no increased risk of overall malignancies in patients with RA compared with the general population, there may be a defined pattern of risk for site-specific malignancies. Based on observed versus expected cases, there was considerable variation in the calculated SIRs among the individual studies for site-specific malignancies. Nevertheless, the random effects meta-analysis demonstrated an overall pattern that was generally consistent with the risk trends reported in the individual studies. This pattern included a clear increase in risks of lung cancer and lymphoma, both Hodgkin and non-Hodgkin, and a potential decrease in risks of colorectal cancer and breast cancer.

The increased risk of lymphoma is especially notable since this malignancy was associated with the highest relative risk, especially for Hodgkin lymphoma, which was more than three-fold higher than in the general population. Although one study suggested a decrease in lymphoma risk (an approximate 50% reduction in non-Hodgkin lymphoma with no reported cases of Hodgkin disease) [6], these results were ascribed to the rarity of these malignancies and the small population that was followed (n = 862), although other studies with similarly small populations (that is, less than or equal to 800 patients) seemed to follow the trends seen in our meta-analysis [10,16,19].

There have been a number of hypothesized explanations for the differences in the risk of certain malignancies in patients with RA compared with patients without the disease. Possible mechanisms for an increased risk of lymphoma in RA patients include the fact that RA results in persistent immunologic stimulation (which may lead to clonal selection and predispose CD5^+ ^B cells to malignant transformation), decreases the number and function of T-suppressor lymphocytes (including those directed against the pro-oncogenic Epstein-Barr virus), and decreases natural killer cell activity in the synovial fluid, tissue, blood, and lymph [24]. Inflammation is believed to play a key role in the risk of lymphoma; epidemiologic studies have suggested that, among patients with RA, higher inflammatory activity is a major risk determinant of lymphoma [25,26]. Meanwhile, the role of RA treatment remains somewhat uncertain; large cohort studies have not confirmed any treatment-related effects; however, it is premature to make conclusions about the risk associated with anti-TNFs with the currently available data [26]. It has been suggested that a minority of RA patients (those with the worst disease) carry much of the increased risk of lymphoma because of their disease rather than their treatment [27].

The observed association between RA and lung cancer may result from several factors. Cigarette smoking would explain an indirect association between RA and lung cancer as smoking is an independent risk factor for both conditions. The direct causal association of RA with lung cancer may be mediated by chronic inflammation and/or the presence of interstitial lung disease. Systemic chronic inflammation has been reported to be a risk factor for lung cancer [28]. A recent 10-year population-based observational cohort study reported that baseline serum C-reactive protein was significantly associated with lung cancer, independent of smoking [29]. In addition, RA has been shown to affect the lungs; autopsy studies have shown some degree of interstitial lung disease in the majority of people with RA [30] and the mortality from pulmonary disease in RA is approximately twice that of the general population [31].

The explanation for the reduced risk of colorectal cancer is most likely due to the increased use of nonsteroidal anti-inflammatory drugs (NSAIDs) and cyclooxygenase-2 (COX-2)-selective inhibitors by patients with RA. These medications have consistently been associated with a decreased risk of colorectal cancer; a recent meta-analysis of all randomized controlled trials and observational studies concluded that COX-2 inhibitors and NSAIDs reduce the incidence of colonic adenomas and that NSAIDs also reduce the incidence of colorectal cancer [32]. The hypothesis underlying this protective association is thought to be the inhibition of COX-2 and subsequently prostaglandin production [33].

The strength of this review is its reliance on real-world clinical data obtained from observational studies rather than randomized placebo-controlled trials that reflect a selected cohort of patients. It provides precise estimates of the malignancy risk in RA patients. However, there are some limitations in the individual studies, as well as with the systematic review and meta-analysis, which should be considered when interpreting the data.

The primary limitation is the heterogeneity among studies in terms of the data sources, populations examined, and study designs. The diversity of study methodologies in some cases may have resulted in bias. Such an example is the study by Mariette and colleagues [15], which was not strictly cohort-derived; it involved identification of new lymphoma cases based on consultation between rheumatology and oncology departments. Uncertainty regarding the size of the RA population evaluated may account for their very high reported relative risk of 7.4 for Hodgkin lymphoma. Sources of selection bias may include the use of hospitalization records for identification of populations.

Other limitations include the possibility of misclassification and the wide variation in follow-up. There may have been misclassification of the inclusion of patients into the RA populations, and there may have been uncertainty surrounding the diagnostic accuracy of the malignancies. Several of the studies were dependent on database analyses and relied on diagnostic codes, whereas another used patient self-report followed by medical record validation. Follow-up times ranged from 1 year to as long as 17 years, and it is possible that in some cases the variability observed in the SIRs may result from these differences. However, these individual study limitations may be compensated for, in part, within the context of performing such a meta-analysis as presented here.

The analysis presented does not attempt to determine causality of risk or adjust for other risk factors that may contribute to the observed increases or decreases in risks, as these data were not readily available in the individual studies. This is especially relevant with respect to severity of disease as well as RA treatment. In relation to treatment effects, nearly all patients in these studies have received treatment for their RA, and it is becoming increasingly likely that treatment of RA is initiated early in the disease process. Consequently, it is difficult to separate the underlying risk associated exclusively with the disease from some of the potential treatment effects, especially when many patients may be taking multiple medications for RA as well as for comorbid conditions. Nevertheless, the consistent findings among the studies included in this meta-analysis where patients were taking diverse medications are consistent with the recent suggestion that it is the underlying inflammation rather than treatment that contributes to the risk [25,26].

## Conclusion

Despite the limitations, a clear trend toward a higher risk of lymphoma and lung cancer was observed in patients with RA. Although a potentially decreased risk of colorectal and breast cancer was identified from the accumulated data in these studies, this observation requires confirmation. Further studies evaluating specific risk factors such as RA management strategies, lifestyle factors, and the presence of the inflammatory process that contributes to RA can help provide additional information on the underlying mechanisms for the observed changes in malignancy risk relative to the general population.

## Abbreviations

CI = confidence interval; COX-2 = cyclooxygenase-2; DMARD = disease-modifying antirheumatic drug; NSAID = nonsteroidal anti-inflammatory drug; RA = rheumatoid arthritis; SIR = standardized incidence ratio; TNF = tumor necrosis factor.

## Competing interests

This study was funded by Bristol-Myers Squibb Company (Hopewell, NJ, USA). SS has been reimbursed less than $10,000 by Bristol-Myers Squibb Company and sanofi-aventis (Paris, France) for honoraria and Scientific Advisory Board Meetings. TAS is an employee of Bristol-Myers Squibb Company. ALS is a consultant of Bristol-Myers Squibb Company and has received more than $10,000 in consulting fees. MCH is a consultant for Bristol-Myers Squibb Company and has received less than $10,000 in consulting fees.

## Authors' contributions

ALS conducted the literature search and helped to draft the manuscript. TAS participated in the design and coordination of the study and helped to draft the manuscript. MCH contributed to the interpretation of the data and helped to draft the manuscript. SS performed the meta-analysis, participated in the evaluation of studies for inclusion, and helped to draft the manuscript. All authors read and approved the final manuscript.

## Supplementary Material

Additional file 1 Table 1. Characteristics of included studies Click here for file
