# Supplementary material for: A meta-analysis of the incidence of malignancy in adult patients with rheumatoid arthritis
Source: Arthritis Res Ther. 2008 Apr 23;10(2):R45. doi: 10.1186/ar2404 (PMC2453765; doi:10.1186/ar2404)
Supplement: Additional file 1 — Table 1. Characteristics of included studies [file ar2404-S1.doc]

| Author, year, country | Data source | Population (N) | Follow-up, years | Malignancies reported (N) |
| --- | --- | --- | --- | --- |
| Abasolo et al.  2007,  Spain [3] | EMECAR cohort | Prevalent RA (789) | Median 3.95; Maximum 6.04 | Overall excluding non-melanoma skin (25)  Non-Hodgkin’s lymphoma (3)  Lung (7)  Colorectal (1)  Breast (2) |
| Askling et al. 2005, Sweden [4,5] | Swedish Inpatient Register, Early Arthritis Register, TNF antagonist register, and other Swedish registers | Prevalent RA (53,067); early RA (3,703); TNF-antagonist treated (4,160) | Mean: prevalent cohort 5.6, early RA 3.6, anti-TNF 2.3; overall range 0-13 | Overall solid (prevalent 3379, early RA 138, anti-TNF 67)  Lymphoma (prevalent 319, early RA 11, anti-TNF 9)  Lung (prevalent 330, early RA 23; anti-TNF 10)  Colorectal (prevalent 342, early RA 18, anti-TNF 10)  Breast (prevalent 471, early RA 13, anti-TNF 8) |
| Cibere et al. 1997, Canada [6] | Rheumatic disease referral center and cancer registry | Prevalent RA (862) | Mean 17.4; range 0.6-35 | Overall (136)  Non-Hodgkin’s lymphoma (3)  Hodgkin’s disease (0)  Lung (16)  Colorectal (10)  Breast (18) |
| Ekstrom et al. 2003, Sweden [7] | National hospital discharge register and other Swedish registers | Prevalent RA (76,527) | Mean 9.6 | Overall (8,898)  Lymphoma (535)  Non-Hodgkin’s (458)  Hodgkin’s (77) |
| Franklin et al.  2006,  United Kingdom [8,9] | Norfolk Arthritis Register | Early RA (1,237) | Mean 7.5 | Overall excluding non-melanoma skin (92)  Lymphoma (9) |
| Geborek et al. 2005, Sweden [10] | Community-based RA cohort, South Swedish Arthritis Treatment Group register, and other population registers | RA cohort without anti-TNF (800); anti-TNF-treated RA (757) | Mean: without anti-TNF 5.5, anti-TNF 2.1; overall range 1.3-5.5 | Overall (without anti-TNF 69; anti-TNF 16)  Lymphoma (without anti-TNF 2; anti-TNF 5) |
| Gridley et al. 1993, Sweden [11] | Swedish Hospital Inpatient Register and other population registers | Prevalent RA (11,683) | Mean 8.6 | Overall (840)  Lymphoma (48)  Non-Hodgkin’s (36)  Hodgkin’s (12)  Lung (68)  Colorectal (72)  Breast (106) |
| Kauppi et al. 1996, Finland [12] | Finnish hospitals’ national discharge registry and other population registers | Prevalent RA (8,920) | Mean 6.9 | Lung (73) |
| Kauppi et al. 1996, 1997, Finland [13,14] | Finnish hospitals’ national discharge registry and other population registers | Prevalent RA (9,469) | Mean 6.9 | Overall (540)  Non-Hodgkin’s lymphoma (34)  Hodgkin’s disease (4)  Colorectal (30) |
| Mariette et al. 2002, France [15] | French rheumatology centers and local oncology departments | Prevalent RA treated with methotrexate (30,000 estimated) | Max 3 | Non-Hodgkin’s lymphoma (18)  Hodgkin’s disease (7) |
| Matteson et al. 1991, Canada [16] | Rheumatoid Arthritis Azathioprine Registry | Prevalent RA treated with DMARDs (530) | Mean 3 | Overall excluding non-melanoma skin (20)  Lung (6) |
| McKendry et al. 1993, Canada [17] | Rheumatic disease clinic records | Prevalent RA treated with methotrexate (144) | Mean 5.6 | Lung (4) |
| Mellemkjaer et al. 1996, Denmark [18] | Danish Hospital Discharge Register and other population registries | Prevalent RA (20,669) | Mean 7; range 1-15 | Overall excluding lymphatic and hematopoietic (1832)  Non-Hodgkin’s lymphoma (85)  Hodgkin’s disease (14)  Lung (308)  Colorectal (204)  Breast (186) |
| Moritomo et al. 1995, Japan [19] | Single health care center and cancer registry | Prevalent RA (655) | Mean 6.1 | Overall (26)  Lung (0)  Colorectal (2)  Breast (3) |
| Setoguchi et al. 2006, United States and Canada [20] | Administrative databases (2 US states, 1 Canadian province) | Prevalent RA treated with biologic DMARDs or methotrexate (7,830) | Mean 4 | Non-Hodgkin’s lymphoma (58)  Lung (169)  Colorectal (118)  Breast (112) |
| Thomas et al. 2000, Scotland [21] | Inpatient records and Scottish Cancer Registry | Prevalent RA (26,623) | Mean 5.7 | Overall (2,029)  Non-Hodgkin’s lymphoma (101)  Hodgkin’s disease (17)  Lung (472)  Colorectal (188)  Breast (249) |
| Wolfe et al.  2007, United States  [22] | National Data Bank of Rheumatic Diseases | Prevalent RA (19,591) | Mean 4.6 | Lymphoma (95) |
| Wolfe et al.  2007, United States  [23] | National Data Bank of Rheumatic Diseases | Prevalent RA (13,869) | Mean 3.5 | Overall excluding non-melanoma skin (543)  Non-Hodgkin’s lymphoma (42)  Hodgkin’s disease (4)  Lung (112)  Colon (37)  Breast (102) |
